# Germination of dimorphic seeds of *Suaeda aralocaspica* in response to light and salinity conditions during and after cold stratification

**DOI:** 10.7717/peerj.3671

**Published:** 2017-08-15

**Authors:** Hong-Ling Wang, Chang-Yan Tian, Lei Wang

**Affiliations:** 1 State Key Laboratory of Desert and Oasis Ecology, Xinjiang Institute of Ecology and Geography, Chinese Academy of Sciences, Urumqi, Xinjiang, China; 2 Central Laboratory, Xinjiang Institute of Ecology and Geography, Chinese Academy of Sciences, Urumqi, Xinjiang, China

**Keywords:** Seed heteromorphism, Light, Cold stratification, Seed germination, Salinity

## Abstract

Cold stratification is a requirement for seed dormancy breaking in many species, and thus it is one of the important factors for the regulation of timing of germination. However, few studies have examined the influence of various environmental conditions during cold stratification on subsequent germination, and no study has compared such effects on the performance of dormant versus non-dormant seeds. Seeds of halophytes in the cold desert might experience different light and salinity conditions during and after cold stratification. As such, dimorphic seeds (non-dormant brown seeds and black seeds with non-deep physiological dormancy) of *Suaeda aralocaspica* were cold stratified under different light (12 h light–12 h darkness photoperiod or continuous darkness) or salinity (0, 200 or 1,000 mmol L^-1^ NaCl) conditions for 20 or 40 days. Then stratified seeds were incubated under different light or salinity conditions at daily (12/12 h) temperature regime of 10:25 °C for 20 days. For brown seeds, cold stratification was also part of the germination period. In contrast, almost no black seeds germinated during cold stratification. The longer the cold stratification, the better the subsequent germination of black seeds, regardless of light or salinity conditions. Light did not influence germination of brown seeds. Germination of cold-stratified black seeds was inhibited by darkness, especially when they were stratified in darkness. With an increase in salinity at the stage of cold stratification or germination, germination percentages of both seed morphs decreased. Combinational pre-treatments of cold stratification and salinity did not increase salt tolerance of dimorphic seeds in germination phase. Thus, light and salinity conditions during cold stratification partly interact with these conditions during germination stage and differentially affect germination of dimorphic seeds of *S. aralocaspica*.

## Introduction

Seed germination is a critical stage in the life cycle of higher plants, especially for annuals (Gutterman, 1993; Gutterman, 2002). In order to germinate at the proper time and suitable place, seeds have evolved a complex suite of mechanisms to integrate environmental signals and regulate the timing of germination in variable field environments (Finch-Savage & Footitt, 2017; Penfield & MacGregor, 2017). Environmental conditions at various life-history stages can influence seed germination (Baskin & Baskin, 2014; Gutterman, 2002). However, seed germination ecology has generally focused on the effects of maternal environment and environmental factors during germination period, while overlooking different environmental variables during cold stratification.

Cold stratification, also known as pre-chilling treatment, is widely used to improve synchronization of germination. It is a requirement of dormancy breaking for seeds possessing physiological dormancy in regions with a cold winter (Baskin & Baskin, 2014). Many studies on cold stratification usually store the dormant seeds on water-moistened substrates in darkness at low temperature (usually between 0 and 5 °C, typically 4 °C) (Noronha, Andersson & Milberg, 1997; Qu et al., 2008; Arc et al., 2012; Einali & Valizadeh, 2017). However, seeds of temperate-zone plants might encounter varying environmental conditions during this cold treatment. Surprisingly, few studies have tested effects of different environmental conditions during cold stratification on dormancy breaking and their interaction with conditions during subsequent germination stage (Baskin & Baskin, 1977; Walck, Baskin & Baskin, 1997; Wang et al., 2011). Seeds on the soil surface can be cold stratified in daily light-darkness cycles, while those buried in the soil will be stratified in continuous darkness. Different light conditions during cold stratification may differentially affect germination. For example, Baskin & Baskin (1977) reported that after one week of stratification, germination percentage of *Asclepias syriaca* was highest for seeds cold stratified in light then incubated in darkness and germination was lowest for seeds stratified in darkness and incubated in light after. In contrast, germination percentages of *Ageratina altissima* achenes were high after 12 wk of cold stratification in light or darkness followed by incubation in light; Germination percentage was only 1–13% after cold stratification and incubation in darkness (Walck, Baskin & Baskin, 1997). Meanwhile, due to environmental heterogeneity, seeds of halophytes in saline deserts of temperate zones may be cold stratified at different salinity levels. Redondo-Gómez et al. (2011) present evidence that, compared with seed germination under different salinity directly, the combinational pre-treatments of cold stratification and salinity decrease germination percentage. Furthermore, although cold stratification is a dormancy-breaking process, non-dormant seeds also might be exposed to cold stratification in temperate regions. Heretofore, no study has compared germination responses of dormant and non-dormant seed morphs of a seed-heteromophic species to different environmental conditions during cold stratification.

Heteromorphic seeds of halophytes in the temperate zone are ideal material to compare the germination responses to cold stratification. Heteromorphic seeds produced by a single plant generally differ in dormancy breaking and germination requirements (Khan, Gul & Weber, 2001; Imbert, 2002; Aguado et al., 2011; Lu et al., 2015; Koen et al., 2017). Based on the classification system for seed dormancy by Baskin & Baskin (2004), two types of dormancy combination for dimorphic seeds of halophytes are recognized: non-dormancy and non-deep physiological dormancy (Wang et al., 2008), and non-dormancy and intermediate physiological dormancy (Wang et al., 2012a). *Suaeda* is a well-known halophytic genus that produces dimorphic seeds, and *S. aralocaspica* (Bunge) Freitag & Schütze is one of the species that produces two seed morphs.

*Suaeda aralocaspica* is an annual halophyte found in inland cold deserts of central Asia (Delectis Florae Reipublicae Popularis Sinicae Agendae Academiae Sinicae Edita, 1979). In China, *S. aralocaspica* is only found in saline desert of the southern margin of the Junggar Basin in Xinjiang Uygur Autonomous Region (Commissione Redactorum Florae Xinjiangensis, 1994). Plants bloom in August and produce dimorphic seeds (non-dormant brown seeds and black seeds with non-deep physiological dormancy) on the same infructescence in September (Wang et al., 2008). Seeds are dispersed in October and germinate in the spring. Thus, seeds can be exposed to cold stratifying conditions during this period. Brown seeds can tolerate high levels of salinity (1,400 mmol L^−1^) during germination; black seeds germinate only under low salinity but still have the vigor after incubation at high salinity (Wang et al., 2008).

The aim of this study was to test the effects of light and salinity conditions during cold stratification and incubation stages on germination of dimorphic seeds of *S. aralocaspica*. We asked the following questions: do brown and black seeds differ in their subsequent germination responses to light and salinity conditions during cold stratification? Does the length of cold stratification change the effect of light and salinity on germination? Are there interactive effects of light or salinity conditions during and after cold stratification?

## Materials and Methods

### Seeds

Freshly matured fruits of *S. aralocaspica* were collected in early October 2011 from a natural population (44°14′N, 87°44′E; 445 m a.s.l.) in saline desert in the northern Xinjiang, China (for a detailed description of this site see Wang et al., 2012b). In the laboratory, dry fruits were manually rubbed to detach the seeds enclosed by the membranous pericarps. Then the brown and black seeds were stored separately at room temperature until used.

### Cold stratification

The following two independent experiments were designed to test the effects of light or salinity during cold stratification on germination. These two experiments were carried out on 18 March, 2012. According to our preparatory experiment, storing fresh matured seeds at room temperature for five months had no significant effect on germination.

Light treatment. Seeds were placed in 5-cm-diameter plastic Petri dishes on two layers of filter paper moistened with 2.5 mL of distilled water. Four replications of 25 seeds of each morph were used per treatment. The lids were sealed with Parafilm to limit water loss during cold stratification and incubation stage. Dishes containing seeds that were to be stratified and/or incubated in total darkness were wrapped with two layers of aluminum foil. All dishes were placed in light (12 h light-12 h darkness cycle, fluorescent lamp, 100 μmol m^−2^s^−1^) for 20 or 40 days at constant 1 °C. A randomized block design was used. Each block consisted of 16 dishes representing a combination of the two seed morphs (brown and black), two light conditions during cold stratification (light or continuous darkness), two light conditions during incubation stage (light or continuous darkness) and two cold stratification treatments (20 d and 40 d).

Salinity treatment. Brown and black seeds were placed separately in 0 (distilled-water control), 200 or 1,000 mmol L^−1^ NaCl solutions at constant 1 °C in continuous darkness for 20 or 40 days. A randomized block design with four replicates was used. Each block consisted of 36 dishes representing a combination of the two seed morphs (brown and black), three salinity during cold stratification (0, 200 or 1,000 mmol L^−1^ NaCl), three salinity during incubation stage (0, 200 or 1,000 mmol L^−1^ NaCl) and two cold stratification treatments (20 d and 40 d). Other procedures were the same as described for light treatment.

### Germination

Light treatment. Following cold stratification, Petri dishes were incubated at daily (12/12 h) temperature regime of 10:25 °C in light or continuous darkness for 20 days. According to the light conditions during cold stratification and germination, there were four treatments: light during cold stratification and germination, light during cold stratification and dark during germination, dark during cold stratification and light during germination and dark during cold stratification and germination. A seed was considered to be germinated when the radicle had emerged ≥5 mm. The number of germinated seeds was counted and germinated seeds were removed from the Petri dishes at the end of cold stratification and incubation period. Germination of seeds both stratified and incubated in darkness was not checked until the end of incubation. We assumed that at the end of cold stratification the seeds had the same germination percentage as those stratified in darkness and incubated in light.

Salinity treatment. Seeds stratified in 0, 200 or 1,000 mmol L^−1^ NaCl solutions were rinsed three times using distilled water, then seeds from each salinity level were transferred to 0, 200 or 1,000 mmol L^−1^ NaCl and incubated at 10:25 °C in light for 20 days. According to the saline conditions during cold stratification and incubation stages, there were a total of nine treatments. Germination was checked before and after the incubation period at 10:25 °C, as described above.

### Statistical analysis

All data were expressed as mean ± s.e. Germination data were arcsine transformed before statistical analysis to ensure homogeneity of variance (non-transformed data appear in both figures). Two- or three-way ANOVA was used to compare treatment effects. LSD test was used to test for differences among treatments when ANOVA showed significant effects (*P* < 0.05). We used nonparametric tests to analyze nonnormal data.

## Results

### Effect of light during cold stratification and incubation stage on germination

**Figure 1:**
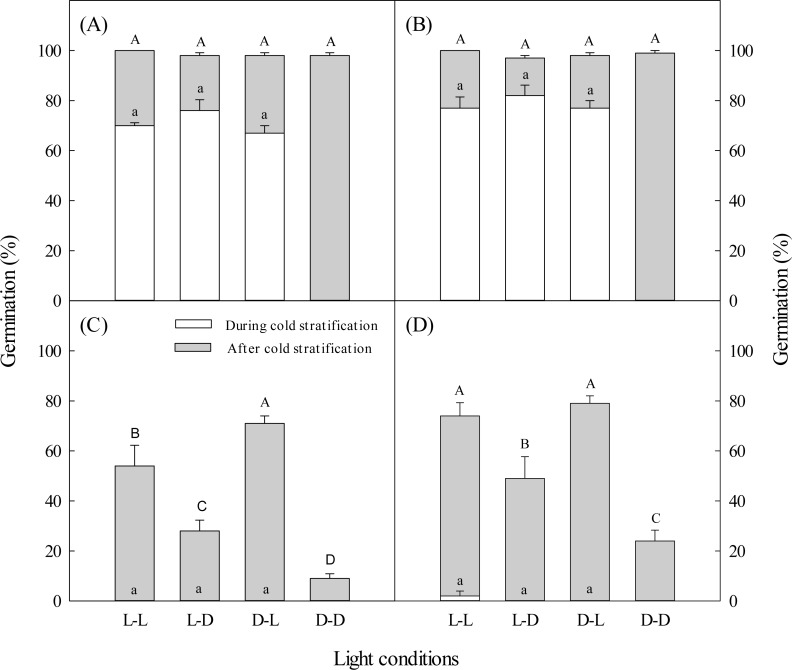
Effects of light during and after cold stratification on germination. Effects of 20-d or 40-d cold stratification in light (L) or darkness (D) on germination percentages of dimorphic seeds of *Suaeda aralocaspica* incubated in L or D at 10:25 °C for 20 d. (A) Brown seeds, 20-d cold stratification. (B) Brown seeds, 40-d cold stratification. (C) Black seeds, 20-d cold stratification. (D) Black seeds, 40-d cold stratification. Significant differences among different light conditions at the end of cold stratification are indicated by different lower-case letters (*P* < 0.05), and at the end of incubation period are indicated by different upper-case letters.

Germination percentage of brown seeds during cold stratification was significantly affected by stratification time (*P* = 0.018) but not by light condition during cold stratification (*P* = 0.189) (Table 1). After incubation at 10: 25 °C for 20 days, total germination percentage of brown seeds was not affected by stratification time (*P* = 1) or by light conditions during cold stratification (*P* = 0.472) and incubation stage (*P* = 0.151) (Table 2). Total germination percentage of black seeds was significantly affected by stratification time (*P* < 0.001), light conditions during germination process (*P* < 0.001) and the interaction between light conditions during and after cold stratification (*P* < 0.001) (Table 1). Black seeds that received a longer stratification period (40 days) showed an increase in ability to germinate in all light conditions (Fig. 1).

**Table 1:** ANOVA of germination conditions on germination. Two- or three-way ANOVA of effect of light conditions during cold stratification and germination stage, the length of cold stratification and their interactions on seed germination of *Suaeda aralocaspica*.

Factor	*d*.*f*.	MS	*P*-value
Germination percentage of brown seed during cold stratification under different light conditions			
Light during cold stratification (LC)	1	46.371	0.189
Stratification time (S)	1	166.162	0.018
LC*S	1	4.396	0.680
Germination percentage of black seed during cold stratification under different light conditions			
LC	1	5.624	0.498
S	1	5.624	0.498
LC*S	1	5.624	0.498
Total germination percentage of black seed under different light conditions			
LC	1	139.921	0.093
Light during germination stage (LG)	1	5525.203	0.000
S	1	894.320	0.000
LC*LG	1	946.096	0.000
LC*S	1	29.528	0.429
LG*S	1	23.507	0.480
LC*LG*S	1	18.197	0.534

**Table 2:** Nonparametric test of germination conditions on germination. Using nonparametric test to analyze the main effects of light or salinity conditions during cold stratification and germination stage and the length of cold stratification on seed germination of *Suaeda aralocaspica*.

Factor	*d*.*f*.	Chi-Square	*P*-value
Total germination percentage of brown seed under different light conditions			
LC	1.000	0.517	0.472
LG	1.000	2.067	0.151
S	1.000	0.000	1.000
Germination percentage of brown seed during cold stratification under different salinity			
Salinity during cold stratification (SC)	2.000	8.773	0.012
S	1.000	0.926	0.336
Germination percentage of black seed during cold stratification under different salinity			
SC	2.000	2.000	0.368
S	1.000	1.000	0.317
Total germination percentage of brown seed under different salinity			
SC	2.000	32.632	0.000
Salinity during germination stage (SG)	2.000	9.700	0.008
S	1.000	0.656	0.418
Total germination percentage of black seed under different salinity			
SC	2.000	9.269	0.010
SG	2.000	36.372	0.000
S	1.000	10.901	0.001

Regardless of whether brown seeds were in light or darkness during the 20-day cold stratification period, more than 67% germinated, but no black seeds germinated (Fig. 1). After 20 days of cold stratification and then 20 days of incubation, cumulative germination of brown seeds was >98% in all light conditions. After 20 days of cold stratification, germination of black seeds was significantly inhibited by darkness, especially when stratified in darkness. Black seeds had the highest germination percentage (71%) when they were cold stratified in darkness and incubated in light. In contrast, 9% of black seeds germinated when they were cold-stratified and incubated in darkness (Fig. 1).

During 40 days of cold stratification in light or darkness, brown seeds germination was >77%, but almost no black seeds germinated (Fig. 1). For 40 days of cold stratification, >97% of brown seeds germinated by the end of the incubation stage. Black seeds showed similar germination responses to light conditions as seeds stratified for 20 days (Fig. 1). Black seeds cold stratified for 40 days had higher germination percentage than those stratified for 20 days.

### Effect of salinity during cold stratification and incubation stage on germination

The percentage of brown seeds which germinated during cold stratification period was significantly affected by salinity (*P* = 0.012) but not stratification time (*P* = 0.336). Total germination percentage of brown seeds was significantly affected by salinity during cold stratification (*P* < 0.001), salinity during incubation stage (*P* < 0.001) but not stratification time (*P* = 0.418) (Table 2). Total germination percentage of black seeds was significantly affected by stratification time (*P* = 0.001), salinity during cold stratification (*P* = 0.01) and incubation stage (*P* = 0.001) (Table 2).

**Figure 2:**
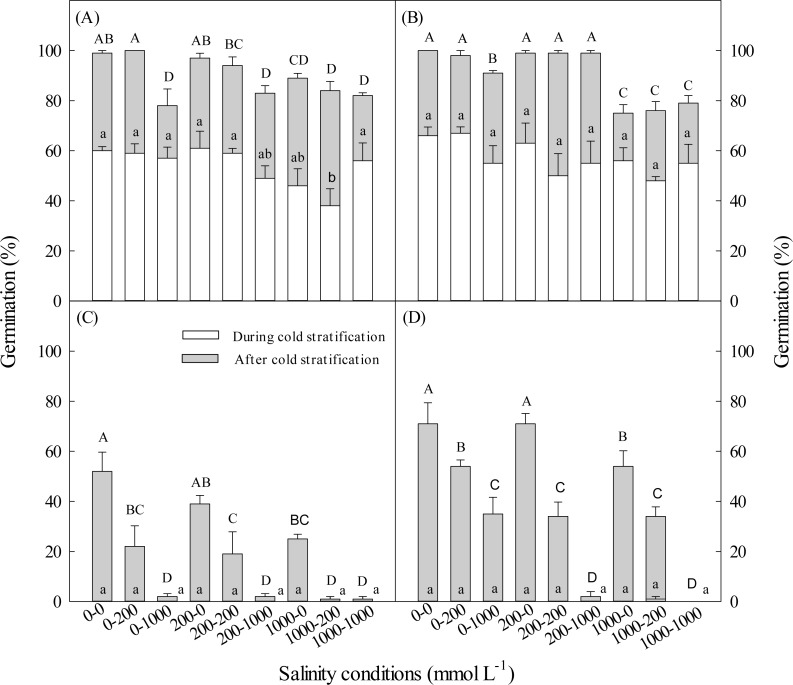
Effect of salinity during and after cold stratification on germination. Effects of 20-d or 40-d cold stratification in 0, 200 or 1,000 mmol L^−1^ NaCl on germination percentages of dimorphic seeds of *Suaeda aralocaspica* incubated in 0, 200 or 1,000 mmol L^−1^ NaCl at 10:25 °C for 20 d. (A) Brown seeds, 20-d cold stratification. (B) Brown seeds, 40-d cold stratification. (C) Black seeds, 20-d cold stratification. (D) Black seeds, 40-d cold stratification. Significant differences among different salinity conditions at the end of cold stratification are indicated by different lower-case letters (*P* < 0.05), and at the end of incubation period are indicated by different upper-case letters.

With the increase of salinity during cold stratification and/or incubation stage, germination percentages of dimorphic seeds decreased. Total germination percentage of brown seeds stratified in 1,000 mmol L^−1^ NaCl was significantly lower than that of seeds stratified in 200 or 0 L^−1^ NaCl (Fig. 2). The longer the cold stratification period, the higher the salt tolerance of black seeds during germination stage (Fig. 2). Cold stratification in salinity did not increase salt tolerance in germinating dimorphic seeds of *S. aralocaspica*.

## Discussion

The effect of cold stratification on seed dormancy and germination has been studied extensively. However, as far as we know, our data are the first to document differences in response of heteromorphic seeds with different dormancy levels to the interactions of different environmental conditions during and after cold stratification. In addition, these data indicate that individual environmental factors during and after cold stratification have a dormancy-specific effect on seed germination. Further, the results show that environmental conditions during cold stratification may not only influence subsequent germination but also interact with the conditions during germination period.

Seeds heteromorphism is a bet-hedging strategy playing a crucial role in the individual survival and population maintenance of plant species with this trait in an unpredictable environment (Imbert, 2002; Lenser et al., 2016). Heteromorphic seeds provide different germination strategies to spread the risk through distribution of plants in time. Different germination requirements of heteromorphic seeds have been observed (Wang et al., 2008; Baskin & Baskin, 2014; Koen et al., 2017). Brown and black seeds of *S. aralocaspica* have distinct germination requirements for stratification time and temperature (Wang et al., 2008). Our result indicated that seed heteromorphism of this species is a complex and elaborate mechanism of germination regulation.

Non-dormant brown seeds and dormant black seeds of *S. aralocaspica* had different germination responses to cold stratification. A high percentage of non-dormant brown seeds germinated during cold stratification, even in high salinity. Cold stratification decreased the germination rate of non-dormant brown seeds of *S. aralocaspica*, but didn’t change the final germination percentage. However, almost no dormant black seeds germinated during cold stratification. Brown seeds began to germinate at low temperature in spring and part of black seeds germinated only after a cold stratification period. Brown seeds do not form a long-lived seed bank in the soil (Wang, 2010). They are dispersed in autumn and either germinate the following spring or die. In contrast, black seeds can form a small persistent seed bank (Wang, 2010). Although germination of cold-stratified black seeds is regulated by light and salinity conditions, cold stratification lowered the light requirement for germination and decreased the inhibitory effect of salinity. The change in germination response may be of adaptive value for dormant black seeds. The possible explanation is that black seeds germinate only if the light requirement at low salinity is fulfilled in the first germination season. If black seeds cannot germinate in the subsequent several germination seasons, they may have no chance to germinate because of the decrease of seed vigor. However, with the prolonged cold stratification period, black seeds obtain the ability to germinate at relatively high salinity in any light conditions.

The response of black seeds of *S. aralocaspica* to light conditions is different from that of *A. syriaca* seeds (Baskin & Baskin, 1977) and similar to that of *A. altissima* achenes (Walck, Baskin & Baskin, 1997). *Suaeda aralocaspica* and *A. altissima* have Type 2 non-deep physiological dormancy (Walck, Baskin & Baskin, 1997; Wang et al., 2008). Cold-stratified black seeds of *S. aralocaspica* required light to germinate to high percentages, but the light requirement could be fulfilled either during the cold stratification or incubation stage. Meanwhile, prolonging the period of cold stratification was effective in reducing the light requirement for germination of black seeds. The responses of dormant seeds to light and cold stratification are related to gibberellin (GA) biosynthesis via regulating GA_3_ oxidase encoded by *AtGA3ox1* or *AtGA3ox2* (Yamauchi et al., 2004; Jiang et al., 2016). However, percentage germination of the non-dormant brown seeds of *S. aralocaspica* was not affected by the light condition during cold stratification or during incubation process. This result probably suggests that the control of bioactive GA levels by phytochrome is not responsible for light-independent seed germination. More research investigating the connection between light condition during cold stratification and germination stages under field conditions is required in order to verify our finding of the interaction of light condition during these two stages.

Salinity in the soil is an important factor influencing germination. Generally, germination of most halophytes is inhibited by high concentration of salinity (Gul et al., 2013). Salinity during dormancy can affect germination of *Suaeda maritima* (Wetson, Cassaniti & Flowers, 2008). Although salinity pre-treatment or cold stratification alone is an effective method to improve salt tolerance of seeds for many plant species, the effectiveness of the combination of these two methods is not certain. For example, cold stratification of seeds using a saline solution as the substrate wetting agent does not have a positive effect for germination and seedling growth of wheat (Wang et al., 2011). Our results showed that cold stratification of seeds with saline solution significantly inhibited germination percentage of dimorphic seeds of *S. aralocaspica*, especially for black seeds stratified at high levels of salinity. The inhibitory effect of cold stratification via salinity was also found in germination of *Cyperus capitatus* seeds at intermediate salinity, although the stratified seeds were not tested at different levels of salinity (Redondo-Gómez et al., 2011). The reason for increasing salt tolerance by cold stratification or salt pretreatment has been discussed in detail (Sharma & Kumar, 1999; Tajdoost, Farboodnia & Heidari, 2007; Iqbal & Ashraf, 2010). However, we could not find a suitable explanation for the lack of promotion effect of cold stratification in salinity. Based on the fact that cold stratification and salt pretreatment are related to biosynthesis of GA (Finch-Savage et al., 2007; Nakaune et al., 2012), further work is necessary to analysis concentration of GA and the expression of related genes during pretreatment and germination periods.

## Conclusions

Non-dormant brown seeds and dormant black seeds of *S. aralocaspica* differentially respond to light and salinity conditions during and after cold stratification. The results suggest that each environmental factor during cold stratification may have the potential to influence subsequent seed germination. Furthermore, environmental conditions during cold stratification may interact with environmental conditions at germination stage. These results are in agreement with Wetson, Cassaniti & Flowers (2008) suggestion that detailed studies of environmental condition during the period from seed dispersal to germination may show it to be more important than is generally suspected. Thus, these should be confirmed by conducting germination tests on seeds with different types of dormancy under various environmental conditions during and after cold stratification in the laboratory and field.

##  Supplemental Information

10.7717/peerj.3671/supp-1 Data S1 Raw data of germination percentage of Suaeda araloscaspica

## Additional Information and Declarations

### Data Availability

The following information was supplied regarding data availability: The germination raw data has been provided as Data S1.
